# Infection and Prophylaxis During Normothermic Liver Perfusion: Audit of Incidence and Pharmacokinetics of Antimicrobial Therapy

**DOI:** 10.1097/TP.0000000000004897

**Published:** 2024-01-10

**Authors:** Saeed Qureshi, Heather Elliott, Alan Noel, Lisa Swift, Corrina Fear, Rachel Webster, Nicholas M. Brown, Rohit Gaurav, Andrew J. Butler, Christopher J. E. Watson

**Affiliations:** 1 The Roy Calne Transplant Unit, Cambridge University Hospitals NHS Foundation Trust, Addenbrookes Hospital, Cambridge, United Kingdom.; 2 Antimicrobial Reference Laboratory, Pathology Sciences Building, North Bristol NHS Trust, Southmead Hospital, Westbury-on-Trym, Bristol, United Kingdom.; 3 Department of Microbiology, Cambridge University Hospitals NHS Foundation Trust, Addenbrookes Hospital, Cambridge, United Kingdom.; 4 The Cambridge NIHR Biomedical Research Centre and the NIHR Blood and Transplant Research Unit in Organ Donation and Transplantation, Cambridge, United Kingdom.

## Abstract

**Background.:**

Ex situ normothermic liver perfusion (NMP) in a blood-based perfusate is associated with a risk of microbe growth, resulting in life-threatening posttransplant sepsis. Antibiotics are widely used, but the pharmacokinetics of these agents are unknown as is their efficacy. We wished to assess the perfusate concentrations of the meropenem and fluconazole that we use and to audit the incidence of infection with this antimicrobial therapy.

**Methods.:**

Fluconazole and meropenem (100 mg each) were added to the perfusate before NMP began, and serial samples were taken and assayed for drug concentrations. Perfusate cultures were available from 210 of the 242 perfusions performed between February 1, 2018, and April 6, 2023; these were reviewed.

**Results.:**

Following administration of 100 mg fluconazole, levels fell slightly from a median of 24.9 mg/L at 1 h to 22.6 mg/L at 10 h. In contrast, meropenem concentrations fell over time, from a median of 21.8 mg/L at 1 h to 9.4 mg/L at 10 h. There were 4 significant microorganisms grown in the perfusions, including 3 *Candida* species and an *Enterococcus faecium*. All the *Candida*-infected livers were transplanted with no adverse consequences, the recipients being treated with anidulafungin upon identification of the infecting organism; the *Enterococcus*-infected liver was not transplanted.

**Conclusions.:**

Serious infection is a risk with NMP but appears to be mitigated with a protocol combining fluconazole and meropenem. This combination may not be appropriate in areas where resistance is prevalent. Routine culture of NMP perfusate is essential to identify breakthrough organisms early and enable recipient treatment.

## INTRODUCTION

Ex situ normothermic machine perfusion (NMP) of deceased donor livers is being increasingly used to extend organ preservation and assess viability.^1-3^ It offers the potential to manipulate perfused organs to improve outcomes, often referred to as resuscitation, reconditioning, or repair. Despite its promise, perfusing an organ at 37 °C in a blood-based medium runs the risk of opportunist infections acquired either from the donor or introduced during perfusion. Initial animal work 20 y ago relied on a second-generation cephalosporin, cefuroxime, to provide antimicrobial cover,^4^ and this prophylaxis has been widely adopted by units starting out on NMP. Cefuroxime has limited antimicrobial efficacy against many organisms, and this lack of efficacy has been associated with life-threatening sepsis in some recipients.^5^ Moreover, it lacks efficacy against *Candida albicans*, a common yeast recognized to cause devastating vascular complications posttransplant.^6,7^

Yeasts and multiresistant organisms are common microbial flora in patients dying in intensive care units exposed to broad-spectrum antimicrobial therapy for several days^8,9^ and commonly contaminate organ transport fluid.^10,11^ The risk of sepsis from contaminating microorganisms during prolonged normothermic liver perfusion with blood-based perfusates has not been established and is likely to be greater than the risk posed by short periods of normothermic kidney perfusion, but even in that setting, infection has been noted.^12^ Hypothermic perfusion is assumed to carry less risk, but cases have been reported after hypothermic oxygenated perfusion of both kidney and liver.^13,14^

We have routinely used a combination of meropenem and fluconazole since acquiring an OrganOx metra liver perfusion machine in 2018. A recent perfusate infection prompted us to review our practice, to undertake pharmacokinetic studies of the antimicrobials we use to assess whether the drug concentrations achieved during perfusion were therapeutic, and to establish their efficacy by reviewing the incidence of significant infection.

## MATERIALS AND METHODS

### Normothermic Machine Perfusion

Livers underwent NMP for donor reasons or logistical reasons related to the recipient operation or operating theater. NMP was undertaken using the OrganOx metra. Perfusate comprised 3 units of packed red cells added to 500 mL of either 5% human albumin solution (HAS, Grifols, United Kingdom) or succinylated gelatin (Gelofusine, BBraun Medical Ltd, Germany), to which were added sodium bicarbonate, heparin, calcium, magnesium, N-acetyl cysteine, and amino acids, together with 100 mg fluconazole (Fresenius Kabi Ltd, Runcorn, United Kingdom) and either 100 or 500 mg meropenem (Bowmed Ibisqus Ltd, Wrexham, United Kingdom). The rationale for this choice of antimicrobial therapy is discussed later. During NMP, livers received continuous infusions of heparin, taurocholic acid, insulin, and epoprostenol. A 20% dextrose infusion was started when the perfusate glucose was <10 mmol/L. The viability assessment to decide whether to transplant the liver followed our previously published criteria.^2,15^

### Recipient Prophylaxis

Recipient antimicrobial prophylaxis comprised piperacillin, tazobactam, and fluconazole given for 5 d. Where the recipient was allergic to penicillin, ciprofloxacin, and metronidazole replaced the piperacillin and tazobactam. If the recipient was a carrier of an extended-spectrum beta-lactamase, they were given meropenem instead. Fluconazole was switched to micafungin for patients with acute liver failure, who underwent retransplants, patients on renal replacement therapy, and those listed on the national “superurgent” waiting list.

### Meropenem and Fluconazole Assays

Samples of perfusate for antimicrobial concentration assays were taken from 13 livers starting before the liver was placed on the machine and then at 1, 2, 4, 6, and 10 h after the start of perfusion. Samples were subjected immediately to refrigerated centrifugation, and the cell-free perfusate was frozen and stored at –20 °C.

Meropenem and fluconazole standard and control powders were procured through Merek: meropenem (PHR1772 and V001253) and fluconazole (PHR1160 and F8929). The high-performance liquid chromatography (HPLC) system comprised a Thermo Fisher Scientific stack: Ultimate 3000 Pump, Ultimate 3000 Autosampler Column Chamber, and an Ultimate 3000 Variable Wavelength Detector. The liquid chromatography mass spectrometry system comprised Shimadzu Pump LC, Shimadzu Auto sampler SIL, Shimadzu System Controller, Shimadzu degassing unit, and PE SCIEX API 3000 LCMS/MS SYSTEM.

Meropenem samples were processed using reverse-phase HPLC using a Hypersil 5 octadecylsilyl 100 mm × 4.6 mm column. Samples underwent a 2-stage process before analysis, first starting with protein precipitation using acetonitrile (1:1 with the sample). This was then mixed and left to stand for 5 min and then microcentrifuged. The supernatant was then extracted, mixed 1:1 with water, and 10 µL was injected into the HPLC system.

The assay was run using an isocratic gradient with a mobile phase containing distilled water, methanol, and orthophosphoric acid (74:25:1).

A calibrator and control were prepared fresh and were either made into HAS or Gelofusine, depending on which sample type was being analyzed. Concentrations of these standards were 100 and 10 mg/L. The lower and upper limits of quantification for the assay were 1.0 and 100 mg/L, respectively; any samples that fell out of the range were diluted accordingly. Blank HAS or Gelofusine samples were performed before analysis to ensure no assay interference.

### Fluconazole

Fluconazole samples were processed using liquid chromatography mass spectrometry with a Synergi Fusion Reverse-Phase 4 µm 80 A column. Sample preparation consisted of prepping the samples 1:2 with acetonitrile spiked with a known concentration of voriconazole D3 (internal standard). This was then mixed and left to stand for 5 min before microcentrifuging. The supernatant was then extracted, and 10 µL injected into the liquid chromatography system.

The assay was run using a Binary Gradient with 2 mobile phases: A phase containing water:methanol:formic acid (90:10:0.1) and B phase containing acetonitrile: methanol: formic acid (90:10:0.1).

A calibration curve with 7 concentrations ranging from 0 to 25 mg/L was made in both HAS and Gelofusine and 3 quality controls (3, 11, and 18 mg/L), and the appropriate type was used for each analysis. Any samples which fell out of this range were diluted accordingly. Multiple reaction monitoring transitions used for analysis were fluconazole 308.20 m/z > 239.10 m/z and for the internal standard (Voriconazole D3) 353.10 m/z > 284.20 m/z. Blank HAS or Gelofusine samples were performed before analysis to ensure no assay interference.

### Review of Infections

Perfusate samples were sent for microbiological culture from all livers at the start and end of NMP. In addition, samples of the cold storage transport fluid were cultured. Culture results were recorded contemporaneously on the electronic patient record for recipients and donors (EPIC Systems, WI), and these results were reviewed.

### Approvals

The perfusate samples used for this study were biobanked as part of a different study with permissions covering further use of samples (Research Ethics Committee reference 21-EE-0177). Assessing the effectiveness of this treatment was considered a service evaluation and, as such, was not considered to require further approval.^16^

## RESULTS

### Pharmacokinetics of Meropenem and Fluconazole

Thirteen liver perfusions were sampled on the basis of having perfusion durations >10 h. Four had Gelofusine and 9 HAS as the plasma substitute. Although the perfusion protocol specified that 100 mg meropenem be given, for 2 of the Gelofusine cases and 2 of the HAS cases the analysis of the perfusate concentrations confirmed that 500 mg had, in fact, been administered.

### Fluconazole

**FIGURE 1.**
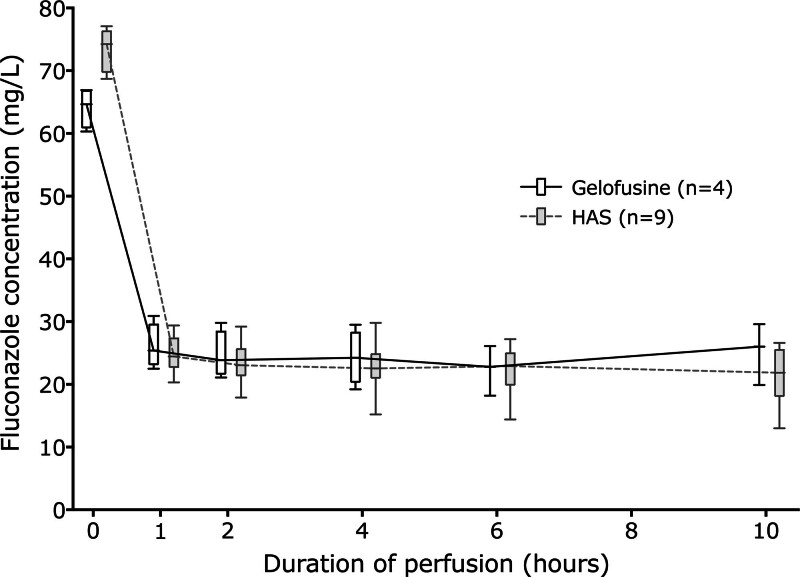
Box and whisker plots of perfusate fluconazole concentration over time following administration of 100 mg fluconazole at time 0 before the liver was placed on the machine.

There was no difference in the perfusate concentrations of fluconazole, whether Gelofusine or HAS had been used (Figure 1). Once the liver was on the machine, the median concentration of fluconazole remained relatively constant throughout the 10-h period, with the median concentration of all 13 perfusions being 24.9 mg/L (range, 20.3–30.9) at 1 h and 22.6 mg/L (range, 13–29.6) at 10 h.

### Meropenem

**FIGURE 2.**
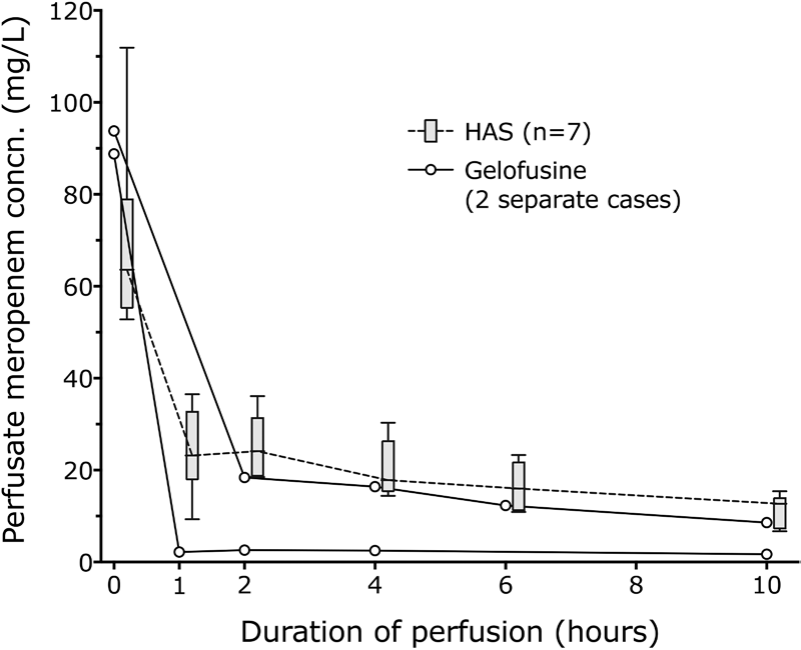
Meropenem concentration over time in perfusate containing HAS (n = 7) or Gelofusine (n = 2, each plotted separately) after an initial dose of 100 mg. HAS, human albumin solution.

**FIGURE 3.**
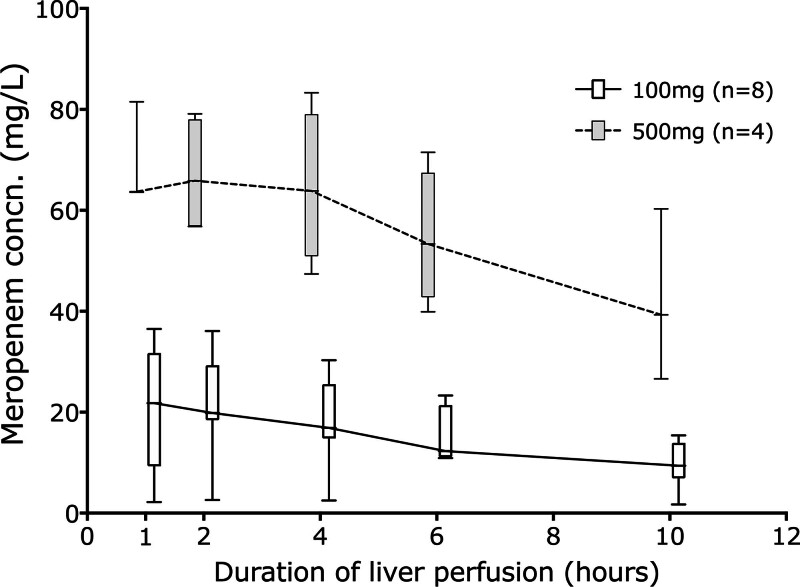
Meropenem concentrations over time after initial dose of 100 and 500 mg.

Two livers received 100 mg of meropenem with Gelofusine, and 2 received 500 mg with Gelofusine. Figure 2 separately shows all livers that received 100 mg meropenem and compares both the 2 Gelofusine livers with the 7 HAS livers. One of the Gelofusine livers had a very low concentration after administration and is discussed later. Figure 3 shows the difference in drug concentrations over time, comparing the 4 livers receiving 500 mg and the 8 receiving 100 mg meropenem.

### Occurrence of Infections

**FIGURE 4.**
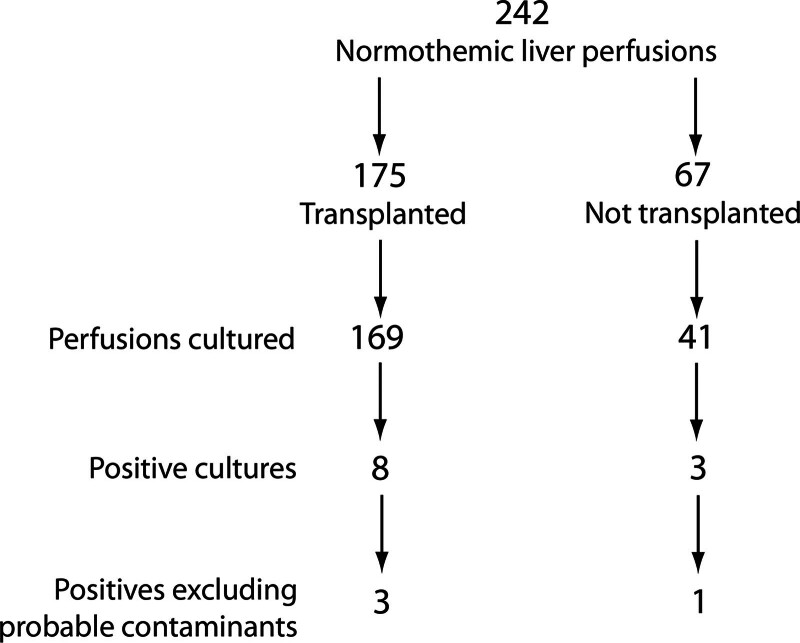
Flow diagram depicting the proportion of normothermic perfusions from which cultures were taken and which were positive.

In the period from February 1, 2018, to April 6, 2023, 242 livers underwent NMP at our institution (Figure 4). Perfusate cultures were available for 210 livers. There were 11 cultures from which 13 organisms were grown. Nine organisms that were grown in 7 perfusions were considered contaminants, being common skin flora and occurring in just 1 of the 2 perfusate cultures; 2 perfusions grew 2 organisms. They included *Staphylococcus epidermidis* (×2), *hominis* (×2), *hemolyticus* (×2), and *warnerii* (×3). From these 7 perfusions, only 4 livers were transplanted. None of the recipients had positive blood or wound cultures postoperatively.

The perfusates from 4 livers had significant growth (Table 1). One perfusate grew *Enterococcus faecium*, an organism that is resistant to meropenem; this liver was not transplanted as the potential recipient died during surgery.

**TABLE 1.** Donor history and culture results for significant infections

Donor age, y	Cause of death	Days on ITU	Donor infections on ITU	Donor antibiotic treatment	Culture of transport fluid	Culture of perfusion fluids	Organism sensitivity	Recipient treatment and outcome
47	Traumatic head injury	3	None recorded	Co-amoxiclav	Not done	*Enterococcus faecium* in end of NMP bottle	Resistant to meropenem	Not transplanted.
43	Respiratory arrest and aspiration	5	*Escherichia coli* in sputum. Aspiration pneumonitis.	MRSA decolonization treatment Co-amoxiclav	*Candida glabrata*	*Candida glabrata*	MIC with fluconazole 32 mg/L	Recipient treated with anidulafungin. No evidence of infection.
47	Traumatic head injury	11	• MRSA in sputum • *Enterococcus faecalis* in urine • *Bacillus cereus* on femoral line	Amoxycillin Metronidazole Temocillin Piperacillin with tazobactam Vancomycin Ceftriaxone	No growth	*Candida glabrata* (only 1 bottle taken)	MIC with fluconazole 1 mg/L	Recipient treated with anidulafungin. No evidence of infection.
52	Hypoxic brain injury secondary to circulatory arrest	2	Basal lung consolidation	Piperacillin with tazobactam	*Candida lusitaniae*	*Candida lusitaniae* (only grew in 1 culture bottle)	MIC with fluconazole 0.25 mg/L	Recipient treated with anidulafungin. No evidence of infection.

ITU, intensive therapy (care) unit; MIC, minimum inhibitory concentration; MRSA, methicillin-resistant *Staphylococcus aureus*; NMP, normothermic liver perfusion.

Three perfusions grew yeast species. One grew a *Candida glabrata* with a minimum inhibitory concentration (MIC) of 32 mg/L with fluconazole. The same organism was present in the University of Wisconsin transport fluid. On day 2, a yeast was identified in the perfusate culture, and prophylactic fluconazole was switched to caspofungin on the assumption that it might be fluconazole-resistant, and then on the following day, switched to micafungin when this was identified as one of the antifungals to which it was sensitive. On day 6, *Candida glabrata* was identified as the organism, and this prompted advice to change the micafungin to anidulafungin.

In a second case, *Candida glabrata* was grown in 1 of the 2 perfusate cultures. It was initially reported as a Gram-positive coccus on day 5 but identified as a yeast on day 6 at which point the recipient’s fluconazole was switched to anidulafungin. It was later confirmed to be a *Candida glabrata* with an MIC of 1 mg/L with fluconazole. There were no infection sequelae.

In the third case, the transport fluid from one of the donor’s kidneys was reported to be growing a *Candida* species on day 2. At the same time, 1 of 2 perfusate cultures was positive, with what was later identified as *Candida lusitaniae*; the same organism grew from a gallbladder swab and in the liver transport fluid. This *Candida* had an MIC with fluconazole of 0.25 mg/L and, as such, was considered sensitive to fluconazole. However, as with the other 2 cases, once notified of a *Candida* species growing and in the absence of full characterization and sensitivities, the recipient was also switched from fluconazole to anidulafungin with no adverse infection sequelae. In none of the 3 cases was the infecting perfusate organism grown in the recipient.

Cultures of the organ transport solution were available for 191 of the perfused livers, of which 28 (14.6%) had positive cultures (Table 2). Only 2 microorganisms identified in the transport fluid cultures were also identified subsequently on perfusate cultures during NMP, *Candida glabrata* and *Candida lusitaniae*.

**TABLE 2.** Organisms detected in positive transport fluid cultures

*Acinetobacter species*	*Cutibacterium acnes ×5*
*Candida albicans*	*Haemophilus influenza*
*Candida glabrata* ^*a*^	*Klebsiella pneumonia*
*Candida lusitaniae* ^*a*^	*Lactobacillus gasseri*
*Candida parapsilosis*	*Providencia rettgeri*
*Citrobacter braakii*	*Pseudomonas extremorientalis/antarctica*
*Citrobacter koseri*	*Pseudomonas tolaasii* ×2
*Coliforms* ×2	*Serratia marcescens*
*Escherichia coli* (ESBL)	*Staphylococcus aureus* (MSSA) ×2
*Escherichia coli* ×2	*Staphylococcus haemolyticus*

a Organism also grew on perfusate during normothermic machine perfusion.

ESBL, extended spectrum beta-lactamase; MSSA, methicillin-sensitive *Staphylococcus aureus.*

## DISCUSSION

In this article we have shown that fluconazole concentrations are stable throughout the 10-h period of liver perfusion studied and are similar whether the perfusate is based on succinylated gelatin (Gelofusine) or 5% HAS. This is in accord with the known predominantly renal elimination of the drug. In contrast, meropenem concentrations fell during the course of perfusion, and long perfusions would require redosing to maintain therapeutic levels. We have also shown that on our protocol of 100 mg meropenem and fluconazole, significant infection during liver perfusion was uncommon, occurring in just 3 of 210 patients (1.4%) and was without clinical significance. The lack of significant infection may reflect our standard antimicrobial prophylaxis posttransplant and prophylaxis during perfusion.

One of the Gelofusine cases receiving 100 mg meropenem had a very low perfusate concentration, reducing from 88.8 mg/L in the perfusate before the liver was added and to 2.2 mg/L at 1 h. The fluconazole level in this same case fell from 60.3 to 30.9 mg/L 1 h after the liver was placed on the circuit. Before a liver is placed on the perfusion machine, it is flushed with 2 L of Hartmann’s Compound Sodium Lactate (Baxter Healthcare Ltd, Thetford, United Kingdom), and some of this remains within the liver and cannulae that dilute the baseline perfusate. With the exception of the unusual Gelofusine case, the median concentration of fluconazole at 1 h was 35% (interquartile range, 33%–36%) of the baseline concentration before the liver was added, and the median concentration of meropenem was 25% (interquartile range, 14%–37%) of the baseline concentration. We cannot account for the single liver with a low meropenem concentration in association with Gelofusine, but this case implies that our initial dose of 100 mg is insufficient to reliably achieve therapeutic drug levels.

The choice of meropenem and fluconazole was based on their antimicrobial spectrum, pharmacokinetic, and pharmacodynamic properties in man, as well as their limited hepatotoxicity. In particular, we wished to cover *Candida albicans* and give broad-spectrum antibacterial cover. We aimed to use drugs with minimal protein binding and minimal partition into red cells because these would contribute to unpredictable pharmacokinetics. Although both meropenem and fluconazole have been associated with raised liver enzymes in patients, we considered them to be the least toxic of the agents we considered with the spectrum of activity we wanted. For example, micafungin, 1 option for anti–Candida prophylaxis, is relatively contraindicated in patients with severe liver function impairment and has been associated with hepatic failure.^17^ Similar concerns exist for anidulafungin,^18^ and even fluconazole has been associated with some hepatoxicity,^19^ but appeared to be the safest antifungal available.

In terms of the dosing, we aimed to achieve concentrations that were in excess of the MIC of common organisms. For fluconazole, 99% of *Candida albicans* were reported inhibited at a MIC of 8 mg/mL, whereas achieving 90% MIC for *Candida glabrata* would require 32 mg/L.^20^ We achieved median levels of 25 mg/L at 1 h and 23 mg/L at 10 h, with the lowest concentration in any of the 13 perfusions being 13 mg/L at 10 h, still efficacious against *Candida albicans*.

For meropenem, the resistance breakpoint for coliforms is >2 mg/L and for pseudomonas is >8 mg/L.^21^ We considered the most concerning bacterial infection to be pseudomonas, which is commonly associated with water and which occasionally contaminates preservation fluids, and therefore considered a target concentration to be >8 mg/L.

Despite our technique, we had 4 infections that we did not consider to be contaminants. Two were *Candida glabrata*, 1 sensitive to fluconazole (MIC 1 mg/L) and 1 resistant to it (MIC 32 mg/L), and 1 was a *Candida lusitaniae* (MIC 0.25 mg/L). The persistence of fluconazole-sensitive *Candida glabrata* and *lusitaniae* in the perfusate may reflect the predominantly fungistatic nature of the drug at the concentrations achieved^22^; neither organism proved clinically significant.

The other infection of note was *Enterococcus faecium*, which is a gut commensal and is often associated with biliary infection.^23^ It is also resistant to meropenem. It occurred in a nontransplanted liver, so the clinical outcome had it been transplanted is unknown. However, during perfusion, there was bile soiling of the perfusate because of insecure cannulation that may account for the perfusate infection. Following this case, our practice has changed, and we now also swab the gallbladder and culture bile during ex situ perfusion. So far, this has yielded 5 positive cultures in 4 of 24 cases, those organisms being the *Candida lusitaniae* previously identified in the perfusate and transport fluid as discussed previously; *Klebsiella pneumoniae* from 2 cases, an *Enterobacter asburiae*, and a *Staphylococcus epidermidis*. Only *Candida lusitaniae* grew in the perfusate

Many more organisms grew in the organ transport fluid, possibly suggesting that the antibiotic prophylaxis had prevented infection with those organisms, with the exception of the fluconazole-resistant *Candida glabrata* and the fluconazole-sensitive *Candida lusitaniae.*

Our antimicrobial protocol was adequate for our practice but may need to be modified elsewhere based on the local prevalence of multiresistant organisms. We believe the use of fluconazole is adequate, given the higher hepatotoxicity of other anticandidal drugs and the low rate of infection. We strongly encourage routine perfusate and bile cultures to get as much warning of significant infections as possible. We have no experience with alternative antibiotics in the perfusate, but if centers wish to use alternative regimens, we recommend they use the same considerations in terms of assessing possible drugs as we describe previously.

## CONCLUSIONS

We have demonstrated that, using appropriate prophylaxis, microbial infection during NMP is uncommon and that perfusate cultures are important to identify serious infections where breakthrough organisms exist. Pharmacokinetics of fluconazole verified that a single dose of 100 mg is adequate and redosing unnecessary, at least for the first 10 h. Meropenem pharmacokinetics suggested that an increased dose of 250 mg may be necessary to be completely assured of appropriate cover and that redosing should be considered at around 10 to 12 h to maintain antibiotic cover.

Local choice of antibiotic should be informed by the prevalence of multiresistant organisms and their sensitivities in the donor population.

Finally, if the recipient of a liver that has undergone a period of ex situ machine perfusion becomes unwell early posttransplant, the possibility of infection derived from the donor and propagated during NMP should be considered.

## ACKNOWLEDGMENTS

The authors are grateful to the liver donors and to the transplant patients who gave consent for samples to be taken that were used in this study.
